# Salivary changes related to systemic diseases in the edentulous patients

**Published:** 2014

**Authors:** E Preoteasa, AM Tâncu, L Iosif, M Melescanu Imre, C Murariu-Măgureanu, CT Preoteasa

**Affiliations:** * Department of Prosthodontics, Faculty of Dental Medicine, “Carol Davila” University of Medicine and Pharmacy, Bucharest; ** Department of Oral Diagnosis, Ergonomics, Scientific Research Methodology, Faculty of Dental Medicine, “Carol Davila” University of Medicine and Pharmacy, Bucharest

**Keywords:** saliva, edentulous, denture, medication

## Abstract

**Introduction:** The relatively frequent systemic comorbidities of geriatric patients can be linked to salivary changes, which may induce oral alteration and discomfort with the removable prosthesis. The aim of the study was to evaluate the salivary parameters in completely edentulous patients treated by removable prosthesis, in relation to their general health status.

**Material and method:** A cross-sectional study was performed on 30 completely edentulous patients, 53% male and 47% female, aged between 53 and 84. The evaluation of the salivary parameters (oral hydration index, pH and salivary flow, viscosity and saliva buffer capacity) was performed with the Saliva Check Buffer kit (GC Corporation).

**Results:** The salivary changes encountered were the following: low hydration level (63%), high saliva viscosity (57%), below-average pH (27%), reduced salivary flow (77%) and low saliva buffer capacity (80%). A reduced salivary flow and saliva buffer capacity was found in women. A lower buffer capacity of the saliva was found in patients with respiratory and gastro-intestinal disease.

**Conclusions:** The alterations of the salivary flow are relatively frequent in geriatric patients, removable denture wearers, with compromised systemic status. These changes may be a risk factor for denture stomatitis and oral candidiasis, with a negative effect on the patient’s comfort and quality of life.

## Introduction

The multiple systemic comorbidities and long-term related medication, that are relatively often encountered in edentulous geriatric patients, with conventional removable dentures or implant supported prosthesis, may be linked to oral health alteration, e.g. xerostomia, oral candidiasis, hyperplasia, denture stomatitis, ulcerations [**1**].

The normal salivary function is an important factor for the maintenance of health, with positive consequences on the functionality and tolerance of the removable dentures [**2**,**3**]. The reduced salivary secretion leads to discomfort in wearing dentures (perceived pain under the dentures), with oral functional impairment (in chewing, deglutition, phonation, as taste alteration), negative influence on the patient’s quality of life [**1**-**3**]. The frequency of the salivary changes in geriatric patients is of 12-28%, being higher in the institutionalized ones - 40-60% [**2**,**4**].

Xerostomia, a subjective symptom consisting in dry-mouth sensation, is frequently associated with quantitative and qualitative changes of the salivary flow. The most frequent causes of xerostomia are the following: medication, primary and secondary Sjögren syndrome, radiotherapy, vasculitis, HIV infections, medullar transplant, and renal dialysis. Medications that most frequently induce xerostomia are anxiolytics, antipsychotics, anticholinergics and antidepressants, diuretics, antihypertensives, sedatives, muscle relaxants, analgesics and antihistamines.

The aim of the study was to evaluate some salivary parameters (oral hydration index, viscosity, pH, salivary flow, and saliva buffer capacity) in completely edentulous patients. At the same time, a stratified analysis of the salivary parameters was performed according to the individual particularities associated with the general status modifications.

## Materials and methods

A cross sectional study was implemented. A convenience sample of completely edentulous patients (in one or both jaws) was formed by patients who requested treatment at the Complete Denture Department, from the Faculty of Dental Medicine, “Carol Davila” University of Medicine and Pharmacy, Bucharest, between 2011 and 2012. The patients have been informed about this study and those who agreed have been included in the sample.

The selection of patients for the study was based on the eligibility criteria detailed as it follows. Maxillary or completely bimaxillary edentulous patients, denture or non-denture wearers, were included in the study. These patients had a modified general status, and, according to the personal declarations, they were under medical treatments with drugs prescribed by the general practitioner. The patients with communication deficiencies and involution phenomena have been excluded from the study in order to avoid errors in data collection. At the same time, patients who could not present a medical letter from their family doctor, which included information about their general status and medication were excluded.

The study variables were the following:

• *Demographic data* - age and sex;

• *General health status* identified diseases were grouped in the following categories: cardio-vascular diseases, diabetes, renal, endocrine, mental and hepatic infections (viral hepatitis), respiratory and gastro-intestinal diseases;

• *Medication *associated to general diseases;

• *Prosthetic status*, evaluated trough the clinical exam, appreciating the edentulous type and prosthetic treatment type;

• *Objective, quantitative and qualitative evaluation of the salivary function*, by using the Saliva Check Buffer (GC) kit. There are 5 steps in these determinations: the first 3 use unstimulated (rest) saliva and the last 2 use stimulated saliva.

Test 1 – Visual inspection of hydration level (low; normal)

Test 2 – Saliva viscosity (increased viscosity, moderate viscosity, normal viscosity)

Test 3 – pH measurement (highly acidic, moderately acidic, healthy saliva)

Test 4 – Saliva quantity (very low, low, normal)

Test 5 – Buffering capacity (very low; low; normal/high)

The data were analyzed by using the SPSS program (Statistical Package for the Social Sciences), version13.00. Mann-Whitney test was used for group comparison.

## Results

The study group consisted of 30 patients, 53% (n=16) male and 47% (n=14) female, aged between 53 and 84 (mean 69 years). Regarding the oral status, 30% (n=9) were completely maxillary edentulous, 20% (n=6) were completely mandibular edentulous and 50% (n=15) were completely bimaxillary edentulous. The patients had numerous systemic diseases. In decreasing the order of their frequency, the identified diseases were the following: cardio-vascular diseases 67% (n=20); diabetes 33% (n=10); gastro-intestinal diseases 20% (n=6); respiratory diseases 17% (n=5); hepatic diseases 17% (n=5); mental diseases 17% (n=5); renal diseases 10% (n=3) and endocrine diseases 10% (n=3).

***Results of the qualitative and quantitative evaluation of the saliva***

According to the tests performed, regarding the objective and qualitative evaluation of the saliva in rest conditions, the results for Test 1 (oral hydration level), showed mostly a decrease of the mucosa hydration in completely edentulous patients. Normal hydration was present only in 37% (n=11), the majority (63%, n=19) presenting a lower hydration.

Regarding the results for Test 2 (saliva viscosity), it was noticed that only 43% (n=13) of the patients presented a normal saliva viscosity, 50% (n=15) having a higher viscosity, and 7% (n=2) had a very high viscosity.

Test 3 (salivary pH evaluation) showed the presence of normal values (73%, n=22) in the majority of patients. 27% presented under average values of pH - 7 patients having a moderate acidity and 1 patient having a higher acidity.

The results obtained in Test 4 (quantity of stimulated saliva), showed important changes of the salivary flow. 40% (n=12) of the patients presented a very low salivary flow, 37% (n=11) a low one and only 23% (n=7) a normal salivary flow.

Test 5 (the buffer capacity of the saliva) showed a decrease of buffer capacity in 80% (n=24) of the patients, with a very important decrease in 30% (n=9) and less important in 50% (n=15). Only 20% (n=6) presented a normal buffer capacity.

Examining the interrelationship of salivary parameters, the following were noticed:

- The patients with a low hydration, compared to those with a normal hydration presented a lower salivary pH, lower salivary flow and lower saliva buffer capacity, the difference being statistically significant, an aspect evaluated with the Mann-Whitney nonparametric test (**Table 1**).

- An increase of the pH was associated (Spearman correlation coefficient = 0.53; p=0.03) with the increase of the salivary flow.

- With the increase of the salivary flow, the buffer capacity of the saliva was also increased (Spearman correlation coefficient = 0.36; p=0.05).

**Table 1:** Interrelation of salivary parameters

**Salivary parameter**	**Subgroups**	**p-value**
pH	Low hydration	<0.001
	Normal hydration	
Salivary flow	Low hydration	<0.001
	Normal hydration	
Buffer capacity	Low hydration	0.05
	Normal hydration	

***Factors involved in the quantitative and qualitative changes of the saliva***

There is a significant statistical difference between the salivary flow between male and female patients from the quantitative point of view and regarding the saliva buffer capacity. Women presented a lower salivary flow and a lower saliva buffer capacity (**Table 2**,**3**). A lower saliva buffer capacity in those with cardio-vascular, gastro-intestinal, respiratory and mental diseases was also noticed. The difference between the groups was statistically significant for the respiratory diseases and marginally statistically significant for the gastro-intestinal ones (**Table 3**). Also, those with cardio-vascular diseases and diabetes had lower levels of pH. The differences between groups were not statistically significant (**Table 4**).

**Table 2:** Comparative evaluation of salivary flow between groups:

**Salivary parameter**	**Subgroups**	**mean**	**p-value**
Salivary flow	male	7.82	p<0,001
	female	2.94	
	the presence of cardio-vascular diseases	5.74	NSS
	the absence of cardio-vascular diseases	5.15	
	with diabetes	5.22	NSS
	without diabetes	5.71	
	with gastro-intestinal diseases	3.75	NSS
	without gastro-intestinal diseases	5.99	
	with respiratory diseases	9.60	NSS
	without respiratory diseases	4.73	
	with hepatic diseases	6.24	NSS
	without hepatic diseases	5.40	
	with mental diseases	5.80	NSS
	without mental diseases	5.49	
	NSS – not statistically significant		

**Table 3:** Comparative evaluation of buffer capacity between groups

**Salivary parameter**	**Subgroups**	**mean**	**p-value**
Buffer capacity	male	9.44	0,02
	female	5.64	
	the presence of cardio-vascular diseases	7.30	NSS
	the absence of cardio-vascular diseases	8.40	
	with diabetes	7.20	NSS
	without diabetes	7.90	
	with gastro-intestinal diseases	7.00	0.079
	without gastro-intestinal diseases	7.83	
	with respiratory diseases	4.60	0.028
	without respiratory diseases	8.28	
	with hepatic diseases	6.80	NSS
	without hepatic diseases	7.84	
	with mental diseases	6.00	NSS
	without mental diseases	8.00	
	NSS – not statistically significant		

**Table 4:** Comparative evaluation of the pH, between groups

**Salivary parameter**	**Subgroups**	**mean**	**p-value**
pH	male	7.14	NSS
	female	6.92	
	the presence of cardio-vascular diseases	6.95	NSS
	the absence of cardio-vascular diseases	7.21	
	with diabetes	6.81	NSS
	without diabetes	7.15	
	with gastro-intestinal diseases	7.00	NSS
	without gastro-intestinal diseases	7.04	
	with respiratory diseases	7.00	NSS
	without respiratory diseases	7.04	
	with hepatic diseases	6.96	NSS
	without hepatic diseases	7.05	
	with mental diseases	6.96	NSS
	without mental diseases	7.05	
	NSS – not statistically significant		

## Discussions

According to the previous results, the alterations of the salivary flow parameters were found in the complete edentulous patients, more severe in women. The most frequent general diseases were the cardio-vascular ones, diabetes and gastro-intestinal diseases. Low hydration of the oral mucosa, a higher saliva viscosity, under average pH, low salivary flow and low buffer capacity were observed as salivary changes. Low values of the salivary flow, under 1ml/min, were present in 73% of the cases, with qualitative alterations of the salivary stimulated and the rest secretion. Regarding the unstimulated saliva secretion, the most significant changes, as decreased levels, were registered in patients with gastro-intestinal diseases, and the buffer capacity was reduced in those with respiratory diseases.

Those who presented a low mucosa hydration, in comparison with those with normal hydration, also had a low pH and a low salivary flow, which explains the higher risk of developing stomatitis, candidiasis and ulcerations in patients with hyposialia.

The study showed that the general complex pathology, with its corresponding medication, might associate alterations to the salivary flow [**3**,**4**]. The decrease of the quantity of unstimulated saliva in patients with diabetes is due to the altered microvascularization and progressive neuropathy [**5**,**6**]. The other researchers reported that low levels of the salivary flow are shown in patients with antidepressant medication, anxiolytics, diuretics and antihypertensive drugs [**7**].

Regarding the implications of the modified salivary flow on the prosthetic treatment, special consideration should be given to aspects related to the denture’s functionality, due to complaints that are frequently related to chewing and device’s retention [**5**]. These aspects can appear due to an insufficient lubrication of the oral mucosa and insalivation of the food bowl [**8**]. Phonation is also affected by the permanent sensation of dry-mouth [**9**]. Hyposialia also frequently associates severe ridge resorption, thus creating bigger difficulties during prosthetic restoration [**10**,**11**]. These aspects sustain the McGill Consensus on nowadays definition regarding the denture standard of the completely mandibular edentulous patients. A more appropriate treatment option is the minimum 2 implants supported overdenture, ensuring an increased denture retention, enhancing the patient’s comfort and quality of life [**10**-**12**].

The “Saliva Check Buffer” kit test from GC allows an objective analysis of the salivary flow. The test is easy and quick to use, giving important information that can help the practitioner in choosing the most appropriate treatment alternative, individualized according to patient’s characteristics. The denture’s materials can be chosen according to their characteristics (e.g., wettability), that might have a positive or negative impact on treatment outcome [**13**,**14**]. The combination of the objective and subjective evaluations offers the practitioner a more clear view during all the prosthetic treatment phases, contributing to the predictability of the treatment outcome [**15**].

## Conclusions

The alterations of the salivary flow, the pH and saliva buffer capacity are relatively frequent in old patients, removable prosthesis wearers, with compromised general status and associated plurimedication. These changes may have implications on oral structures, their functional and support capacities, on the dentures’ balance and tolerance, may be a risk factor for prosthetic stomatitis, oral candidiasis and some traumatic injuries caused by the dentures. The “Saliva Check Buffer” test kit can be used in the edentulous patients with conventional dentures or implant supported overdentures, giving information regarding the parameters of the salivary flow. Using this knowledge gives the dentist the possibility to adopt the appropriate measures for the prevention of the negative effects associated with the salivary changes and to improve the dentures functionality, reduce discomfort and improve the patient’s quality of life.

## References

[1] Preoteasa E (2005). Aspecte clinice şi terapeutice la edentaţi total cu modificări ale statusului oral.

[2] Preoteasa E, Ionescu E, Bencze A, Preoteasa CT (2006). Considerations regarding the treatment with total dentures for patients with hyposalivation. A clinical case. Rev Med Chir Soc Med Nat Iasi.

[3] Nederfors T (2000). Xerostomia and Hyposalivation. Adv Dent Res.

[4] Negrato CA, Tarzia O (2010). Buccal alterations in diabetes mellitus. Diabetol Metab Syndr.

[5] Wolff A, Gadre A, Begleiter A (2003). Correlation between patients satisfaction with complete dentures and denture quality, oral condition, and flow rate of submandibular/sublingual salivary glands. Int J Prosthodont.

[6] Guggenheimer J, Moore AP (2003). Xerostomia- Etiology, recognition and treatment. JADA.

[7] Bergdahl M, Bergdahl J (2000). Low unstimulated salivary flow and subjective oral dryness: association of medication, anxiety, depression and stress. Journal of Dental Restoration.

[8] Iosif L, Preoteasa E, Murariu-Magureanu C (2010). Protetical stomatitis and general diseases interrelationship. Literature review. Revista de Medicina Militara.

[9] Preoteasa E, Preoteasa CT, Melescanu-Imre M, Iosif L, Tancu AM, Sultan AN (2011). Influenta statusului general asupra starii de sanatate orala la edentatul total. Revista de Medicina Militara.

[10] Melescanu-Imre M, Preoteasa E, Buzea MC, Preoteasa CT (2009). Implant based overdenture - a piece within an ethical domino. Romanian Journal of Bioethics.

[11] Melescanu Imre M, Marin M, Preoteasa E, Tancu AM, Preoteasa CT (2011). Two implant overdenture - the first alternative treatment for patients with complete edentulous mandible. Journal of Medicine and Life.

[12] Preoteasa E, Meleşcanu-Imre M, Preoteasa CT, Marin M, Lerner H (2010). Aspects of oral morphology as decision factors in mini-implant supported overdenture. Rom J Morphol Embryol.

[13] Preoteasa CT, Sultan AN, Popa L, Ghica MV, Ionescu E, Tâncu AMC, Preoteasa E (2011). Studies regarding the wettability of acrylic and silicone dental materials. Farmacia.

[14] Preoteasa E, Iosif L, Amza O, Preoteasa C, Dumitrascu C (2010). Thermography, an imagistic method in investigation of the oral mucosa status in complete denture wearers. J Optoelectron. Adv. Materials.

[15] Preoteasa E, Imre M, Preoteasa CT (2014). . A 3-Year Follow-up Study of Overdentures Retained by Mini-Dental Implants. Int J Oral Maxillofac Implants.

